# Electrochemical and Friction Characteristics of Metallic Glass Composites at the Microstructural Length-scales

**DOI:** 10.1038/s41598-018-19488-7

**Published:** 2018-01-17

**Authors:** Aditya Ayyagari, Vahid Hasannaeimi, Harpreet Arora, Sundeep Mukherjee

**Affiliations:** 1 Department of Materials Science and Engineering, University of North Texas, Denton, Texas 76203 USA; 2 Department of Mechanical Engineering, School of Engineering, Shiv Nadar University, Uttar Pradesh, 201314 India

## Abstract

Metallic glass composites represent a unique alloy design strategy comprising of *in situ* crystalline dendrites in an amorphous matrix to achieve damage tolerance unseen in conventional structural materials. They are promising for a range of advanced applications including spacecraft gears, high-performance sporting goods and bio-implants, all of which demand high surface degradation resistance. Here, we evaluated the phase-specific electrochemical and friction characteristics of a Zr-based metallic glass composite, Zr_56.2_Ti_13.8_Nb_5.0_Cu_6.9_Ni_5.6_Be_12.5_, which comprised roughly of 40% by volume crystalline dendrites in an amorphous matrix. The amorphous matrix showed higher hardness and friction coefficient compared to the crystalline dendrites. But sliding reciprocating tests for the composite revealed inter-phase delamination rather than preferred wearing of one phase. Pitting during potentiodynamic polarization in NaCl solution was prevalent at the inter-phase boundary, confirming that galvanic coupling was the predominant corrosion mechanism. Scanning vibration electrode technique demonstrated that the amorphous matrix corroded much faster than the crystalline dendrites due to its unfavorable chemistry. Relative work function values measured using scanning kelvin probe showed the amorphous matrix to be more electropositive, which explain its preferred corrosion over the crystalline dendrites as well as its characteristic friction behavior. This study paves the way for careful partitioning of elements between the two phases in a metallic glass composite to tune its surface degradation behavior for a range of advanced applications.

## Introduction

Metallic glass composites (MGCs) developed over the last decade promise a unique combination of toughness and tensile ductility unachievable in amorphous materials^1,2^. Metallic glass composites can be broadly classified into *ex situ* and *in situ* composites. *Ex situ* composites, comprising of tungsten, steel, or carbon fibers as the reinforcing phase, suffer from a number of limitations including interface de-bonding, stress concentration, and limited wetting of reinforcement during thermoplastic forming^3–5^. In contrast, *in situ* crystalline phase reinforced metallic glass composites do not show many of these limiting characteristics. Several MGCs exhibit as high as 10% tensile ductility unlike bulk metallic glasses that typically show brittle failure under tensile loading and low fracture toughness^6,7^. These alloys have been reported to have excellent mechanical properties in quasi-static and dynamic loading^8^, at supercooled liquid temperatures^9^, and show good fatigue^10^ and fracture properties^11,12^. The crystalline dendrites impart ductility and toughness by arresting unlimited extension of shear bands in the amorphous matrix^13^. Potential applications for MGCs include high-performance sporting goods, bio-implants, spacecraft gears, and many other military and industrial goods which demand high hardness, good corrosion resistance, large elastic strain and thermoplastic processing ability for complex geometries^14^. Monolithic metallic glasses, with fully amorphous structure, typically show good corrosion resistance because they do not have grains and grain boundaries, which act as pit nucleation sites in crystalline materials. In addition, they have excellent wear resistance because of high surface hardness. However, there is limited understanding of the corrosion and wear behavior of metallic glass composites^15–17^. The effect of chemistry and phase-specific mechanical properties on the overall surface degradation characteristics of MGCs has not been reported. Fundamental understanding in this regard will lead to better design of metallic glass composites by controlling the relative fraction and chemistry of the constituent phases.

Here, we report on the electrochemical and friction characteristics of a metallic glass composite at the microstructural length-scale and correlate with the bulk wear and corrosion behavior. Zr_56.2_Ti_13.8_Nb_5.0_Cu_6.9_Ni_5.6_Be_12.5_ alloy was chosen because of its distinct microstructure and commercial importance. Bulk wear was evaluated by sliding reciprocating tests and corrosion behavior was assessed through potentiodynamic polarization. Phase-specific tribological tests included nano-indentation, nano-scratch and scanning vibration electrode technique (SVET). To understand the friction behavior at the microstructural length-scale, *in situ* scratch data was obtained for each phase concurrent with direct observation inside a scanning electron microscope. Relative work-function difference between the two phases, measured by scanning kelvin probe (SKP) microscopy, was used to explain the overall surface degradation behavior.

## Experimental

The alloy was prepared by vacuum arc melting high purity starting elements in Ti-gettered argon atmosphere. The cast alloy was placed in a copper boat encapsulated in Ar filled quartz tube and heated using induction technique^1^. The alloy was heated to about 825 ± 25 °C, and held isothermally for several minutes^1^. This was followed by quenching the alloy to obtain two-phase microstructure. For subsequent characterization, samples were extracted from the cast-ingots in the dimensions of 10 mm length, 8 mm width and 3 mm thickness. Cut metallic glass composite samples were polished to 1200 grit finish according to ASTM standard E3-11. Microstructural characterization was done using FEI Quanta environmental scanning electron microscope (SEM). Nano-indentation using Hysitron Triboindenter TI-750 L (Hysitron, Inc., Minneapolis, MN USA) determined the phase-specific hardness and modulus. Nano-indentation recipe consisted of 5 s loading, 2 s holding and 5 s unloading cycles with a diamond Berkovich tip at 10,000 µN load. Indentations were done in a square matrix of 5 × 5 with a spacing of 25 µm between the indents. Five such indentation matrices were created to cover a large area comprising of both phases. Nano-scratch was done at 3000 µN load with a displacement of 10 µm to evaluate phase-specific coefficient of friction. Ten scratches were done to quantify the run-to-run variation. PI88 SEM Picoindenter (Hysitron, Inc., Minneapolis, MN USA) was used to directly observe the phase-specific scratch behavior inside a FEI Quanta dual beam focused ion beam (FIB) microscope. A diamond cube-corner tip was used to create scratch tracks near dendrite-matrix interfaces. 3000 µN load was applied to create five 15 µm long scratch tracks at a rate of 500 nm/s. Since the experiments were carried out inside a high-vacuum SEM chamber, the influence of surface oxidation, contamination and ambient moisture is likely to be small.

Bulk wear tests were done using Rtec Universal Tribometer (Rtec Instruments, San Jose, CA, USA). A 3 mm WC ball was used as counterface. Tests were performed at Hertzian contact pressures of 1.8 GPa and 2.1 GPa, 5 Hz frequency of reciprocation and stroke length of 1 mm. The tests were run from 1 m to 15 m (corresponding to duration of 2 to 20 min) with incremental steps of 1 m (2-minute interval) in lab air (≈47% RH). Optical interferometry was used to generate wear track profile and Gwyddion data visualization and processing software to quantify the wear volume loss.

Electrochemical studies were performed using Gamry Ref 3000 Potentiostat and a three-electrode cell comprising of a high density graphite plate as the counter electrode, saturated calomel electrode (SCE) as the reference electrode, and the MGC sample as the working electrode. The sample was exposed via a 6 mm diameter orifice to 1 M NaCl electrolyte. Open circuit potential (OCP) was noted until fluctuations were less than 0.200 V/min. Potentiodynamic polarization was carried out from 0.25 V below stable OCP at a scan rate of 0.167 mV/s. The test was terminated after pitting, identified as a current surge in the system.

Site-specific electrochemical behavior was evaluated by Princeton Applied Research scanning electrochemical microscope (SECM). Samples for SKP and SVET were cold-mounted to fit micro-cell dimensions and polished to 0.1 micron surface roughness. Dry tests were performed first, after which the cell was filled with 1 M NaCl electrolyte and SVET was carried out. Scanning kelvin probe (SKP) module was used for relative work function measurement over an area of 50 µm^2^. A tungsten probe was used as a reference during SKP measurements. Work function was determined at a working distance of 100 µm for all experiments, with dry lab air forming the dielectric between the probe and sample.

SVET analysis was conducted with VersaSCAN micro-scanning electrochemical workstation with vibrating Pt/Ir probe of diameter ~10 µm. The probe movement was controlled with a piezoelectric motion controller over the XY plane. Stable OCP was ensured by running a potential-time (E-t) test with PARSTAT potentiostat. All the SVET tests were carried out at no external bias. A sample area of 0.1 mm × 0.1 mm was scanned for SVET analysis with a step size of 5 µm/s at a time constant of 20 ms. The vertical amplitude of the probe vibration was set to 30 µm normal to surface with a frequency of 80 Hz and the data were collected via a lock-in-amplifier attached to the system. The probe to sample distance was maintained at 100 µm. The SVET area scans were recorded at the beginning of the test and after every 24 h for four days.

## Results and Discussion

### Wear Rates and Volume Loss

**Figure 1:**
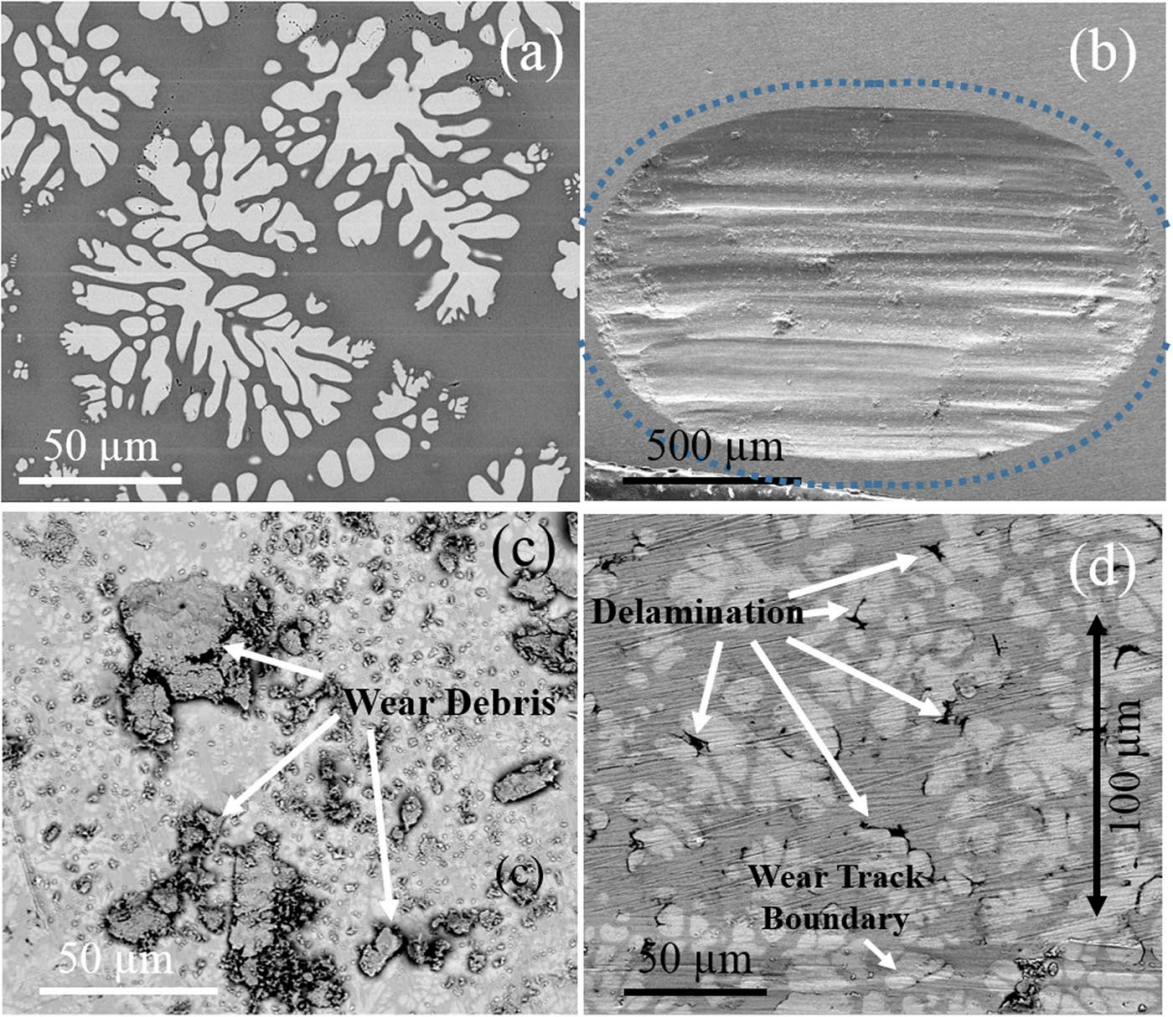
(**a**) Representative back scattered electron micrograph of MGC; (**b**) representative wear track after 20 min of sliding at 10 N load. The region where there was interphase delamination has been marked with the blue dotted line as a guide to the eye; (**c**) wear debris formed due to sliding wear at 10 N load (**d**) interphase delamination between the amorphous and crystalline phases at the periphery of the wear track. The dendrites appear brighter than the matrix due to partitioning of heavier elements into the crystalline phase. Wear track shows uniform deformation on surface. There was no preferred wearing of one phase over another. Wear debris did not show any indication of oxidation.

An SEM image of the composite is shown in Fig. 1a. The nominal composition of the alloy was Zr_56_Ti_14_Nb_5.0_Cu_7_Ni_6_Be_13_, with amorphous matrix composition of Zr_47_Ti_13_Nb_7_Cu_21_Ni_13_ and *in situ* crystalline dendrite composition of Zr_72_Ti_9_Nb_14_Cu_2_Ni_2_. Be could not be detected by energy dispersive x-ray spectroscopy (EDS). The relative fraction was ~40% crystalline dendrites and ~60% amorphous matrix. The crystalline phase had a higher amount of Zr and Nb, while the amorphous matrix had more of Ti, Cu and Ni. SEM image of a representative wear track at the end of 15 m sliding with 10 N load is shown in Fig. 1b. The morphology and wear features were very similar for 5 N load (not shown). Bulk deformation under a spherical indenter showed uniform and simultaneous wearing of both phases. This observation is in contrast to a previous study on Ti-based MGC where preferred adhesive wear of the crystalline dendritic phase was seen^18^. No preferred adhesion of either of the two phases was observed in the present study on Zr-based MGC. Wide and deep (approximately 15–20 µm) grooves formed due to ploughing parallel to the sliding direction. The wear particles had a flake-like appearance and were comprised of both amorphous and crystalline phases as seen from the Z-contrast SEM image in Fig. 1c. There was no evidence of particle adhesion to the counterface or wear tribofilm transfer, both of which may be consequences of rub-off from repeated sliding. The counterface surface remained intact with no visible deformation. These features point towards abrasion as the predominant mechanism of material removal. Figure 1d shows inter-phase separation at the periphery of the wear tracks, which is a signature of plastic deformation. Such delamination extended outward to about 100 µm from the edge of the wear track, beyond which the material remained intact. EDS was performed on the wear track and wear debris to identify oxidation/oxidative wear. Spot scan spectra revealed very little oxidation of the wear debris and negligible oxidation on the wear track. This minimal oxidation may be attributed to the relatively low temperature rise during sliding reciprocation, in line with earlier reports^17^.

**Figure 2:**
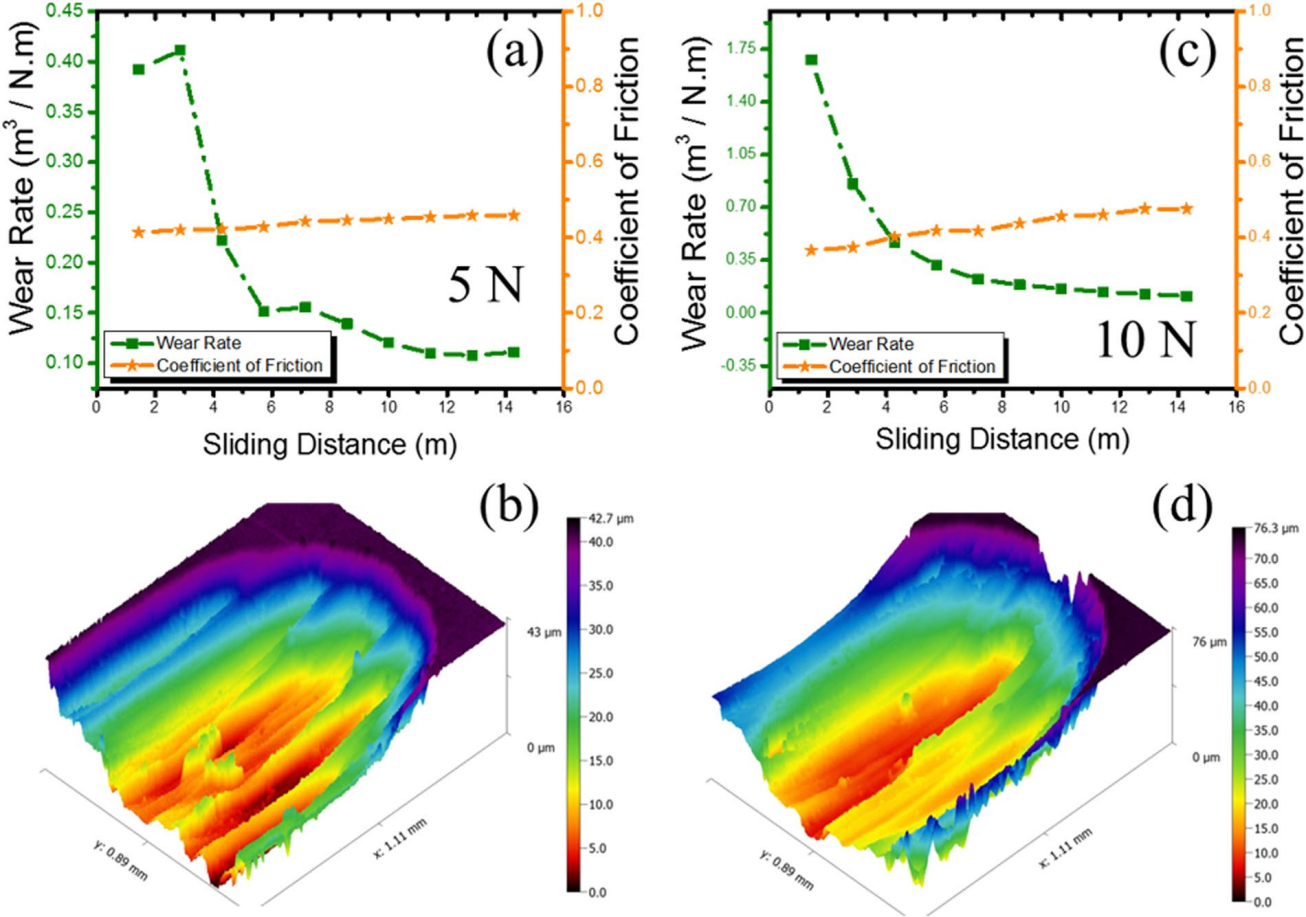
Bulk wear results showing the (**a**) wear rate and coefficient of friction for 5 N load and (**b**) corresponding 3D optical profiler image at the end of test; (**c**) wear rate and coefficient of friction for 10 N load and (**d**) corresponding 3D optical profiler image. Wear volume loss in the 10 N test condition was significantly higher than the 5 N test condition, although friction values were similar.

The wear rate, coefficient of friction and optical interferometry image of wear track for 5 N load are shown in Fig. 2a and b. The friction values observed in the current set of experiments are comparable to the ones reported previously^19^. Corresponding data and image for 10 N load are shown in Fig. 2c and d. The wear rate for both test conditions dropped continually with increasing sliding distance. The wear rate and volume loss for 10 N were significantly higher than 5 N test condition, in line with previous reports^19^. The depth of wear track was about 42 µm for the 5 N test condition, and 75 µm for 10 N load. The mean coefficient of friction value was similar for both test conditions.

### Phase Specific Hardness and Friction

**Figure 3:**
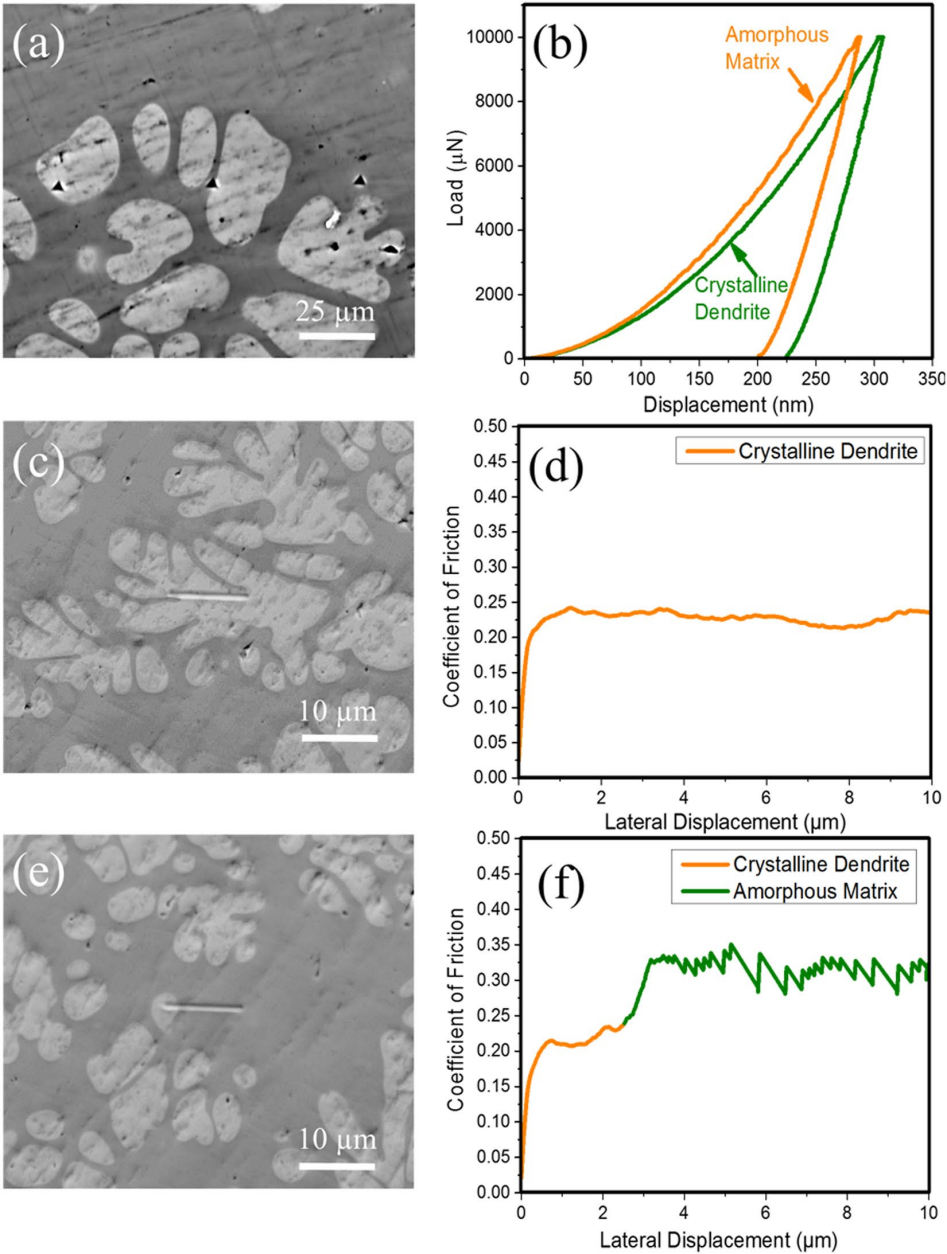
Phase-specific nano-indentation results: (**a**) SEM image showing indentations on bright crystalline dendrite and dark amorphous matrix and (**b**) load versus displacement curves obtained on the respective phases; (**c**) SEM image of scratch test performed on crystalline dendrite and (**d**) the corresponding coefficient of friction; (**e**) Scratch on both crystalline and amorphous phases and (**f**) the corresponding coefficient of friction for both phases. The friction coefficient was higher for the amorphous matrix and had a saw-tooth type profile compared to a smooth/uniform value for the crystalline dendrite.

Nano-indentation and nano-scratch were done on the amorphous matrix and crystalline dendrite individually to obtain phase-specific surface properties. Representative indentations on the bright crystalline dendrite, dark amorphous matrix, and the interface are shown in Fig. 3a. The corresponding load displacement curves are shown in Fig. 3b. The average hardness of dendrite and amorphous matrix were found to be 5.98 and 7.14 GPa respectively. The standard deviation was less than 6% in each case. The modulus was 105 ± 18 GPa for the crystalline dendrite and 115 ± 7 GPa for the amorphous matrix. The hardness/modulus (H/E) ratio was calculated to be 0.054 for the dendrite and 0.062 for the matrix. H/E ratio is an indicator of elastic to plastic transition, which was higher for the amorphous matrix compared to the crystalline phase. A material with higher H/E ratio transitions from elastic to plastic behavior at higher stress levels compared to those with lower H/E ratio. The crystalline phase with lower H/E likely deformed plastically compared to elastic deformation for the amorphous phase resulting in interface cracking as seen in the bulk wear tests. The scratch morphology on the dendrite is shown in Fig. 3c and the corresponding friction profile in Fig. 3d. The scratch shown in Fig. 3e initiates on the crystalline phase and extends to the amorphous matrix. The coefficient of friction was lower for the crystalline phase (~0.22) as compared to the amorphous matrix (varying between 0.27 and 0.35) as shown in Fig. 3f. The saw-tooth friction profile for amorphous matrix is attributed to shear banding and prow formation as the indenter deformed the matrix^18,20,21^.

**Figure 4:**
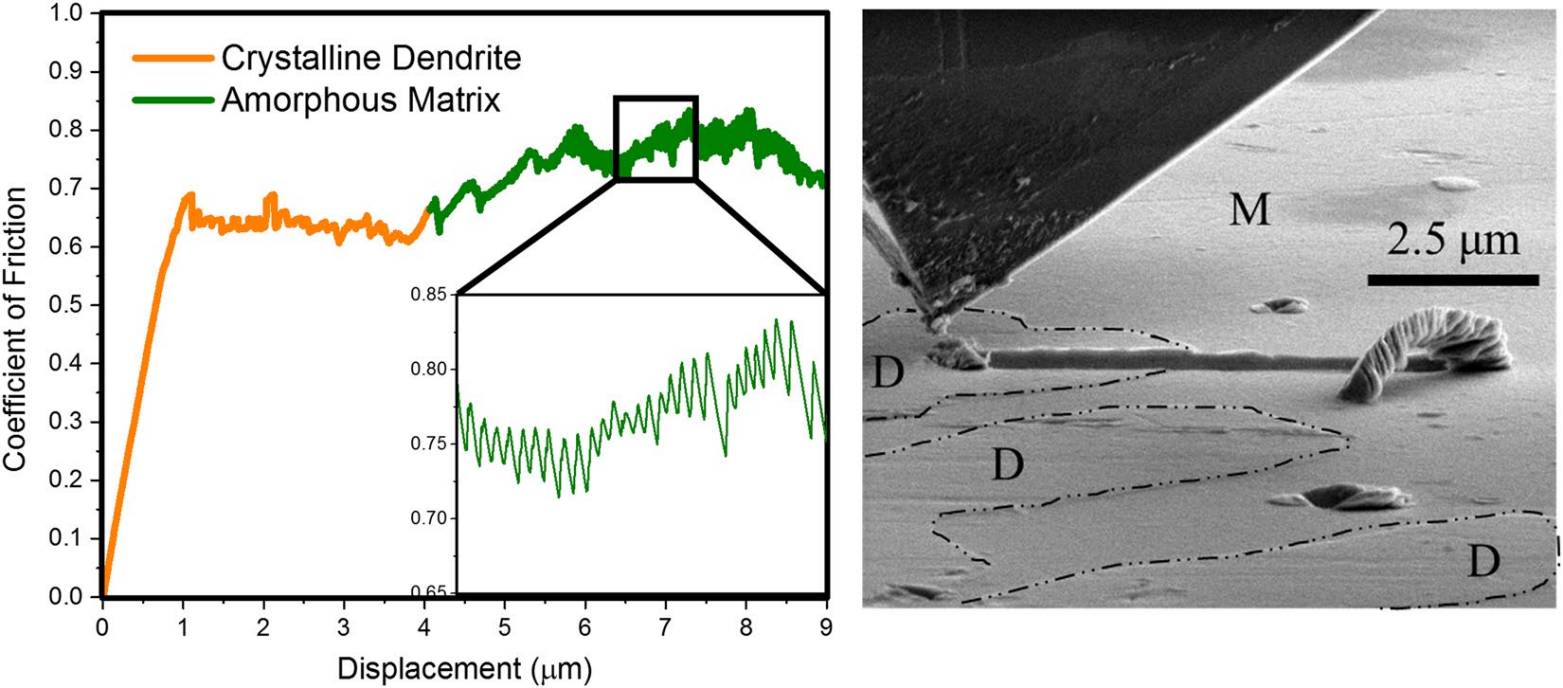
(**a**) Coefficient of friction measured using pico-scratch on the interface of dendrite and matrix material. A sharp rise in the friction value is seen on the interface as the indenter transitions from dendrite (denoted with D) to matrix (denoted with M). (**b**) SEM image of the sample and pico-indenter after competition of scratch test. The dendritic phase is discontinuous as highlighted with dash-dot line. The cube corner diamond indenter has retracted to the initial position after traversing the set scratch length. There is no material adhering to the indenter, and the displaced material is ejected out of the wear track, and dislodged at the edge.

Phase-specific scratch behavior was studied using PI88 SEM Picoindenter concurrent with direct observation of material removal mechanism. Figure 4a shows the friction coefficient across the interface of the two phases. Corresponding SEM image of the scratch track is shown in Fig. 4b. The average friction value for the dendrite (marked with D on Fig. 4b) was ~0.65. There was a sharp increase in friction value across the interface. The average friction coefficient for the amorphous matrix was 0.8 (marked with M on Fig. 4b). This is similar to the behavior observed in nano-scratch (Fig. 3c). The friction values using the cube-corner probe in PI88 Picoindenter was higher than the nano-scratch test done using Berkovich probe. However, the trend in friction remained similar – matrix showed higher friction value compared to the dendrite. Consistent with the nano-scratch test, there was no material/prow adhering to the indenter as seen in the SEM image (Fig. 4b). Instead, the material removed from the scratch track ejected out and formed a pile at the end of the track. This indicates that adhesive behavior may not be active in the tribosystem. The material pile-up dislodged at the end of the scratch track had multiple folds. This may be attributed to stick-slip type behavior. A magnified view of friction values for the amorphous matrix is shown in the inset of Fig. 4a, which is similar to the saw-tooth profile seen during nano-scratch test. These features are clearly seen in the supplemental video.

### Corrosion and Polarization Behavior

**Figure 5:**
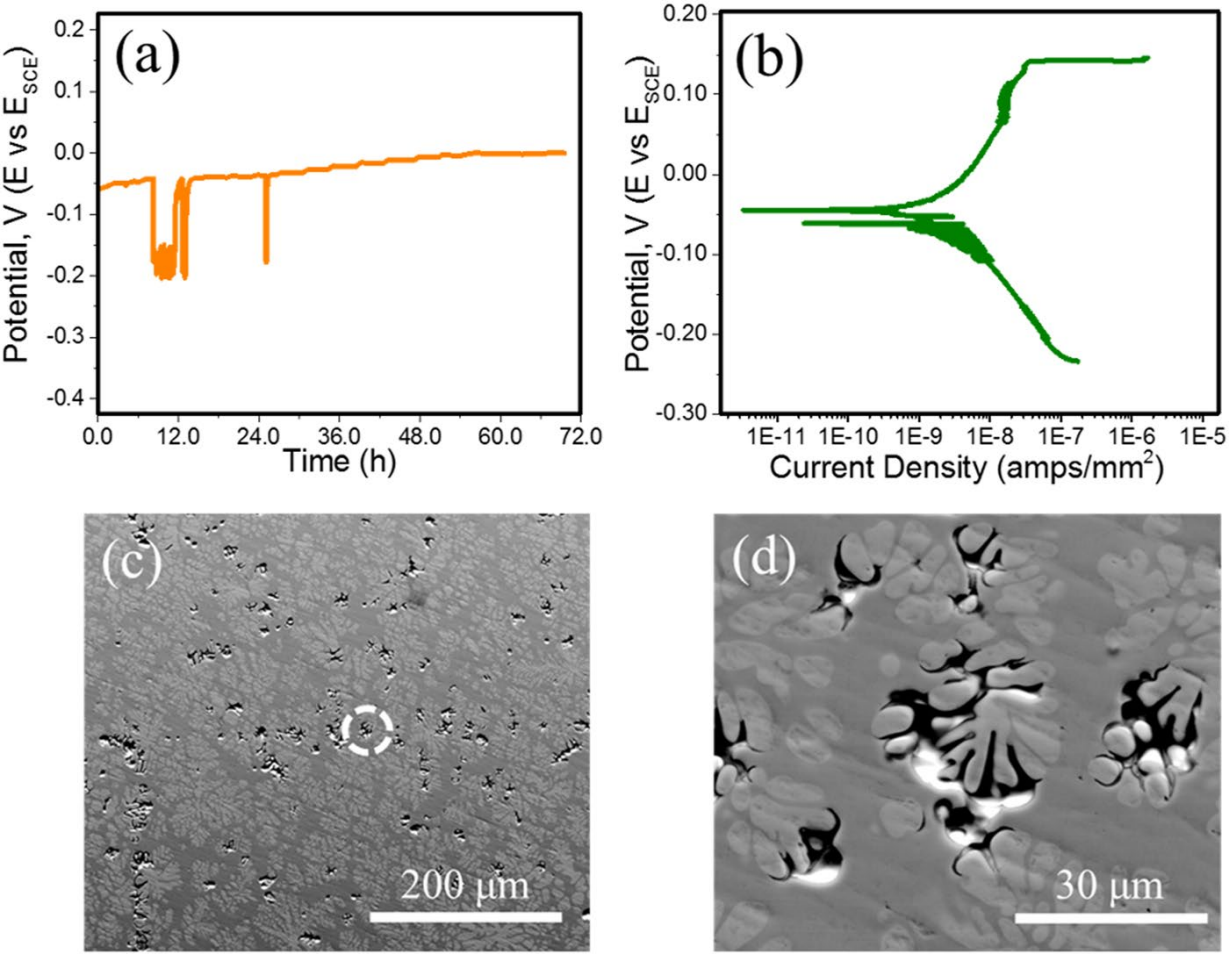
(**a**) Open circuit potential for the MGC in 1 M NaCl solution for a 72 h test; (**b**) potentiodynamic polarization curve for MGC; (**c**) low magnification SEM image showing the density and distribution of pits on the metallic glass composite; (**d**) high magnification image of the inset highlighting interface corrosion due to galvanic coupling. Transient potential drops seen in the open circuit potentiogram may be attributed to the transient anodic sites forming on the surface.

The open circuit potential in 1 M NaCl solution for the MGC is shown in Fig. 5a. OCP was measured for an extended period until the rate of change of potential was almost negligible indicating the formation of a stable passive layer. OCP for the MGC showed instantaneous passivation in contact with the NaCl electrolyte. This was followed by spikes in potential, which are related to the breakdown and re-passivation of the surface^22–24^. The passive layer broke down after 12 hours of exposure seen as a potential drop in Fig. 5a. After this, the sample continually oscillated between passivation and rupture until the potential rose back to the initial OCP value corresponding to re-passivation of the surface. The re-passivized sample did not show any potential transients after 24 hours until the test was terminated at 72 hours. Potentiodynamic polarization was performed after the sample attained stable OCP. Multiple short current spikes evident on the cathodic branch of the polarization curve (Fig. 5b) likely resulted from galvanic coupling between the amorphous and *in situ* crystalline phases in the alloy. In a separate set of experiments, it was observed that holding the sample in the potential range of −100 to −150 mV (vs SCE) resulted in preferential dissolution of the amorphous matrix between the dendrites. The amorphous phase is less noble compared to the *in situ* crystalline dendrite phase. Therefore, small part of the polarization curve (Fig. 5b) is anodic for the amorphous phase while it is cathodic for the dendrite. This resulted in galvanic coupling. Pitting was seen at ~0.140 V (vs SCE) and a current density of 33 × 10^−9^ amps/mm^2^. The current value continued to surge until 1.73 × 10^−6^ amps/mm^2^. The pitting morphologies after potentiodynamic polarization test are shown at low and high magnification in Fig. 5c and d respectively. Pitting was mainly seen at the interphase boundary confirming that galvanic coupling was the predominant corrosion mechanism. In addition, there was preferential corrosion of the amorphous matrix in between the dendrites. This may be attributed to the unfavorable ratio of anode to cathode sites. A small anodic region trapped between highly cathodic sites is depleted more rapidly. Rather remarkably, no depletion/pitting for the crystalline dendrite phase was observed on the entire sample. This was investigated further by phase-specific electrochemical tests.

### Phase Specific Electrochemical Characterization

**Figure 6:**
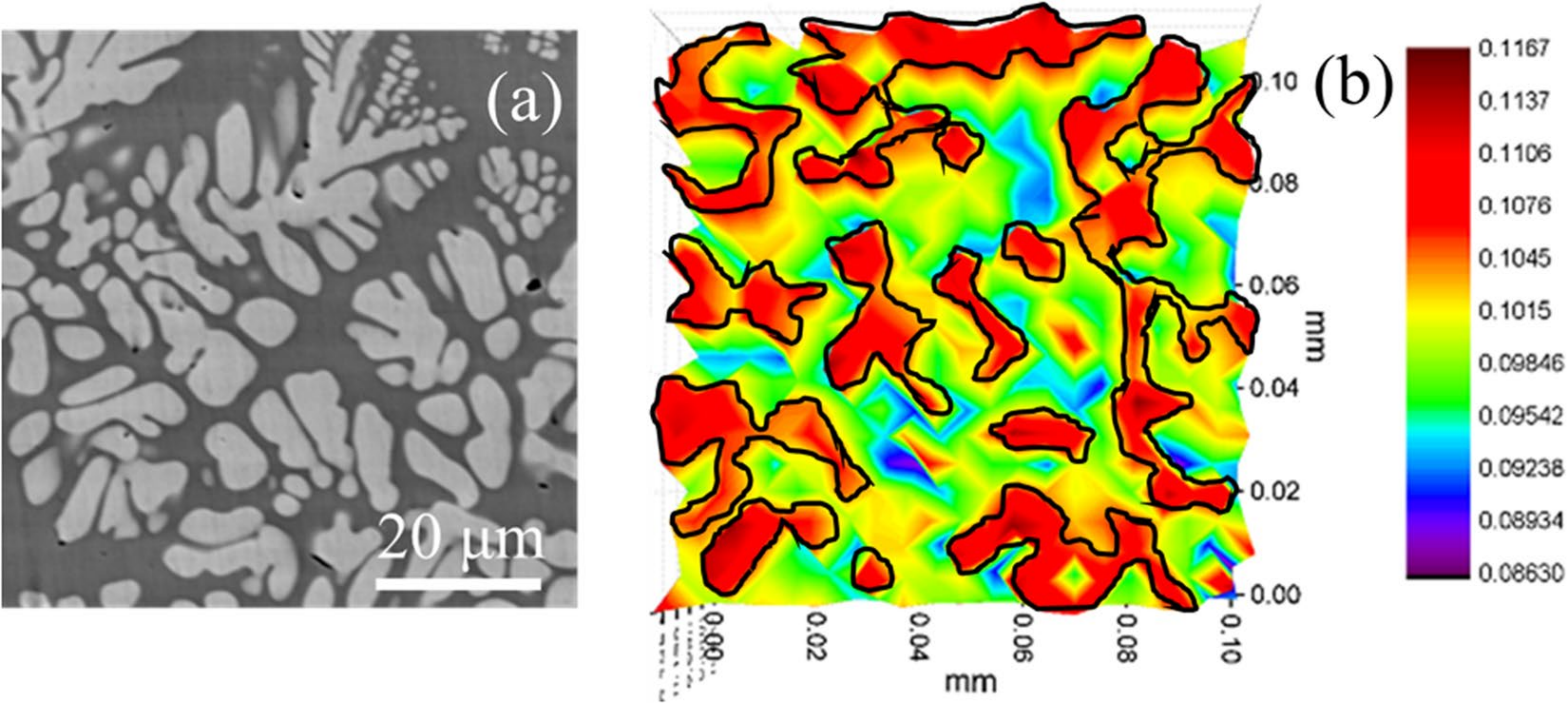
(**a**) Representative microstructure of MGC surface scanned by scanning kelvin probe (SKP) for relative work function mapping; (**b**) variation in relative work function measured over 100 µm square area. The discontinuous regions (colored red) correspond to the crystalline phase while the continuous region (colored yellow to blue) corresponds to the amorphous matrix.

The sample surface was scanned by SKP microscopy to create a relative work function map. A representative microstructure and work function map at the same microstructural length-scale are shown in Fig. 6a and b, respectively. The discontinuous regions in the work function map (shown in red) represent the crystalline dendrites in the contiguous amorphous matrix (yellow-blue). The crystalline dendrite showed higher relative work function compared to the amorphous matrix. A strong correlation between corrosion potential and work function has been reported for crystalline^25,26^ and amorphous materials^27^. Typically, alloys with higher work function show better corrosion resistance due to lower electropositive behavior. Therefore, amorphous matrix with lower work function has higher electropositivity compared to the crystalline dendrite. This may also explain the scratch behavior. More electropositive metallic alloys show higher adhesion for metal-on-metal wear compared to alloys that are relatively passive. The higher electropositive nature of the amorphous matrix may have resulted in greater stiction with the indenter and more localized stresses. Buckley’s hypothesis^28,29^ suggests that chemically active metals (electron donors) are prone to strong adhesion compared to inert metals even at low loads thus resulting in strong stiction and higher frictional resistance^30^.

**Figure 7:**
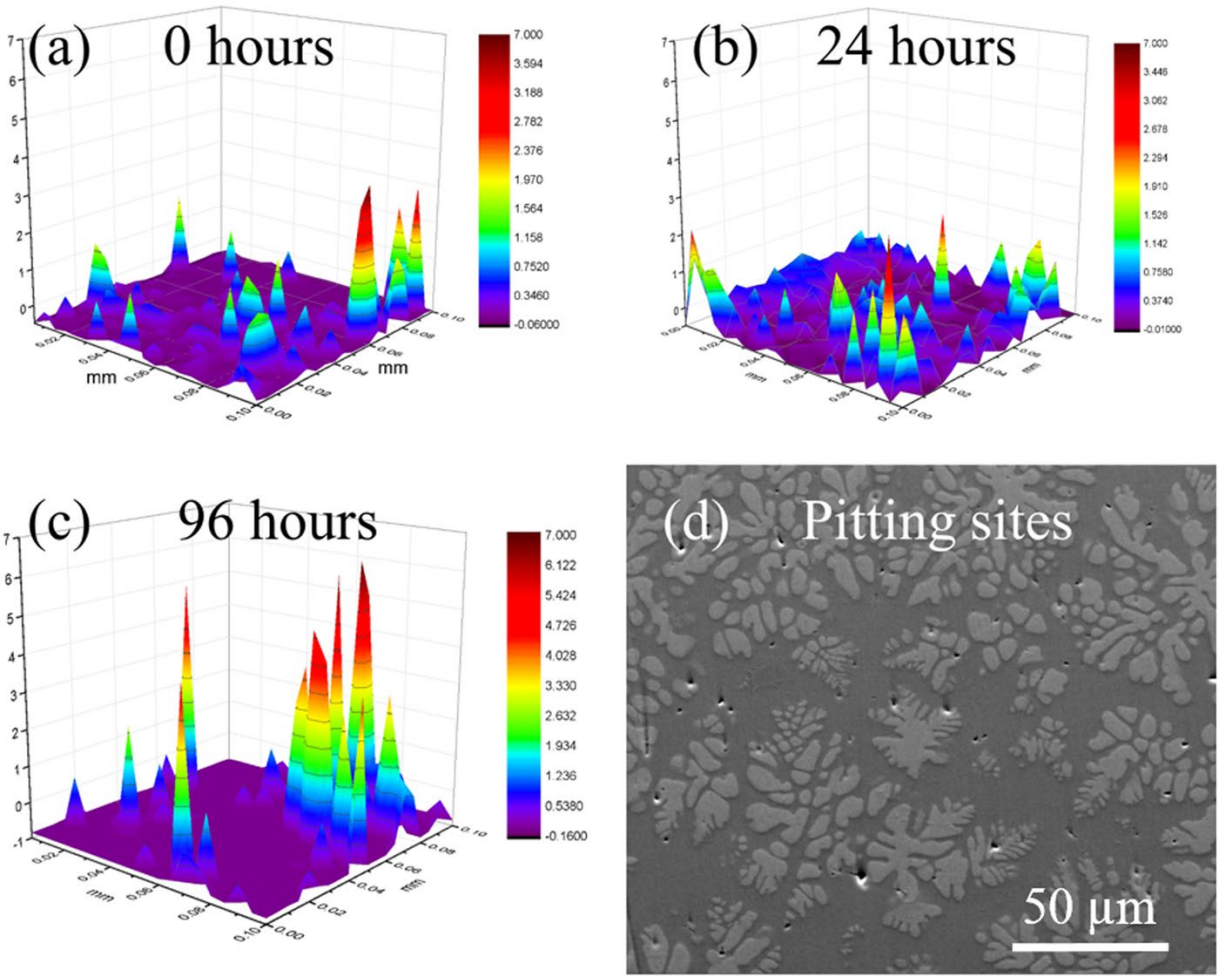
Potential variation measured by scanning vibration electrode technique (SVET) at (**a**) the start of test, (**b**) 24 hours of immersion, and (**c**) 96 hours of immersion in 1 M NaCl; (**d**) topography contrast image after 96 h immersion from SVET test showing uniform corrosion of the matrix and consequent embossed type appearance of the dendrite and pitting at the inter-phase boundaries.

SVET measurements were performed in NaCl solution to determine change in potentials on the surface in contact with electrolyte. The corrosion potential distribution on the alloy surface after stabilization is shown in Fig. 7a. The high and low potential regions represent active and neutral electrochemical sites, which may further develop into pit initiation or transient anodic sites. These sites may grow into pits or may passivate with time^31^. Potential variation with time was mapped after different time intervals. The 3D potentiogram after 24 hours of immersion is shown in Fig. 7b at the same scale as the starting potential map. The number density of anodic sites increased, but there was decrease in peak intensity. This indicates the active nature of the surface with simultaneous initiation of new anodic sites, passivation of previously formed sites and increase in anodic potential of few others. The potentiogram after 96 h (Fig. 7c) of immersion is significantly different from the 0 h and 24 h plots. The complete disappearance of some peaks that formed at the 24-hour mark indicates passivation. Increased potential value of pre-existing peaks may be related to higher electrochemical activity of stable anodic sites. Figure 7d shows SEM image of the sample after 96 h exposure to the electrolyte. The SEM micrograph confirms that two corrosion mechanisms occur simultaneously: (i) uniform dissolution of amorphous matrix but not the crystalline dendrites and (ii) majority of pits forming at the inter-phase boundary.

Uniform dissolution of amorphous matrix and consequent embossed type relief of the dendrites is shown in Fig. 7d. Similar corrosion susceptibility was reported for a Ti-based metallic glass composite tested in different electrolytes^32,33^. The composition of the amorphous matrix was found to be Zr_46.8_Ti_12.6_Nb_6.5_Cu_20.8_Ni_13.2_ and that of the crystalline dendrite was Zr_72.4_Ti_9.4_Nb_13.8_Cu_2.4_Ni_2.0_. A micro-capillary flow injection inductively coupled plasma mass spectroscopy technique was used to estimate the relative rate of dissolution of elements from bulk metallic glasses. Cu was found to have the highest dissolution rate followed by Ni. The dissolution rate was lowest for Nb followed by Zr^34^. The crystalline phase in the MGC is enriched with slow dissolving elements, Nb (13.8% vs 6.2% in the matrix) and Zr (72.4% vs 46.8% in the matrix). In contrast, the amorphous matrix contains higher fraction of rapidly dissolving elements, Cu (20.8% vs 2.4% in the dendrite) and Ni (13.2% vs 2.0% in the dendrite). Be distribution studied by secondary ion mass spectrometry (SIMS) and electron energy loss spectroscopy (EELS) has been reported in a recent study^35^. *In-situ* dendrites in Be-bearing MGCs were reported to be Be-free. The Be almost entirely partitioned to the glassy matrix. Since Be is highly reactive, it is expected to dissolve with the higher percentage of less noble elements like Cu and Ni in the glassy matrix. The preferred dissolution of matrix over dendrite in this *in situ* composite may be attributed to this difference in chemistry.

Majority of the pitting sites after 96 h SVET were found to be at the inter-phase boundaries (Fig. 7d). These sites are prone to corrosion due to the sharp composition variation across the phase boundary along with the difference in crystal structure. The observations in SVET match well with the OCP data in terms of multiple transients of corrosion and re-passivation over an extended period. SKP shows the amorphous matrix to be more electropositive which explains its preferred corrosion over the dendrite. Accelerated corrosion tests that reveal alloy depletion around the dendrite is in line with the work function observations. SKP also explains the friction behavior. The stiction and saw-tooth COF seen in nano-scratch test on the amorphous matrix is in line with its higher electropositivity.

## Conclusions

Phase-specific electrochemical and friction behavior was evaluated for fundamental understanding of the overall surface degradation characteristics of metallic glass composites. Bulk wear tests showed inter-phase delamination, which was attributed to the difference in hardness/modulus (H/E) ratio of the two phases and elastic to plastic transition at different stress levels. Faster and uniform dissolution of the amorphous matrix in the composite during corrosion test in 1 M NaCl electrolyte resulted in embossed type relief of the crystalline dendrites. This was attributed to the difference in chemistry, the amorphous matrix having higher fraction of rapidly dissolving elements compared to the crystalline dendrites. Phase chemistry affected the electronic structure in terms of the amorphous matrix showing more electropositive behavior in scanning kelvin probe microscopy. Stick-slip type interaction with the tip during scratch test and larger coefficient of friction further validated the higher electropositivity of the amorphous matrix. In summary, appropriate partitioning of elements between the two phases in a metallic glass composite maybe used as a toolbox for tuning its electrochemical and friction characteristics for a range of advanced applications including spacecraft gears, high-performance sporting goods and bio-implants.

## Electronic supplementary material

Video 1
